# A realist systematic review of evidence from low- and middle-income countries of interventions to improve immunization data use

**DOI:** 10.1186/s12913-021-06633-8

**Published:** 2021-07-08

**Authors:** Allison L. Osterman, Jessica C. Shearer, Nicole A. Salisbury

**Affiliations:** PATH, 2201 Westlake Avenue, Suite 200, 98121 Seattle, WA USA

**Keywords:** Data use, Data quality, Health information system, Routine data, Immunization data

## Abstract

**Background:**

The use of routine immunization data by health care professionals in low- and middle-income countries remains an underutilized resource in decision-making. Despite the significant resources invested in developing national health information systems, systematic reviews of the effectiveness of data use interventions are lacking. Applying a realist review methodology, this study synthesized evidence of effective interventions for improving data use in decision-making.

**Methods:**

We searched PubMed, POPLINE, Centre for Agriculture and Biosciences International Global Health, and African Journals Online for published literature. Grey literature was obtained from conference, implementer, and technical agency websites and requested from implementing organizations. Articles were included if they reported on an intervention designed to improve routine data use or reported outcomes related to data use, and targeted health care professionals as the principal data users. We developed a theory of change *a priori* for how we expect data use interventions to influence data use. Evidence was then synthesized according to data use intervention type and level of the health system targeted by the intervention.

**Results:**

The searches yielded 549 articles, of which 102 met our inclusion criteria, including 49 from peer-reviewed journals and 53 from grey literature. A total of 66 articles reported on immunization data use interventions and 36 articles reported on data use interventions for other health sectors. We categorized 68 articles as research *evidence* and 34 articles as *promising strategies*. We identified ten primary intervention categories, including electronic immunization registries, which were the most reported intervention type (*n* = 14). Among the research evidence from the immunization sector, 32 articles reported intermediate outcomes related to data quality and availability, data analysis, synthesis, interpretation, and review. Seventeen articles reported data-informed decision-making as an intervention outcome, which could be explained by the lack of consensus around how to define and measure data use.

**Conclusions:**

Few immunization data use interventions have been rigorously studied or evaluated. The review highlights gaps in the evidence base, which future research and better measures for assessing data use should attempt to address.

**Supplementary Information:**

The online version contains supplementary material available at 10.1186/s12913-021-06633-8.

## Background

Within global health, it is widely acknowledged that a cornerstone of well-functioning health systems are sufficiently high-quality data to guide decision-making around health service delivery. Calls to improve the quality and use of data feature prominently in several national plans of action and in global strategies like the Global Vaccine Action Plan. Donor bodies including The Global Fund to Fight AIDS, Tuberculosis and Malaria; US President’s Emergency Plan for AIDS Relief (PEPFAR); and Gavi, the Vaccine Alliance; among others, have also identified data quality and data use as strategic focus areas. While investments in national health information systems and advances in information technology have improved the timeliness, quality, and availability of health data, data remain an underutilized resource in decision-making, especially at the level of health care delivery [1, 2]. In the immunization sector, data use is recognized as lacking in the design and implementation of programs, leading to calls for more evidence regarding effective strategies to improve data use [3].

Although the barriers to using health data have been relatively well studied and point to insufficient skills in data use core competencies among health workers, lack of trust in data due to poor quality, and inadequate availability because of fragmented data across multiple sources, among others [1, 4–8], to date there is no formal review of evidence from existing efforts to strengthen immunization data use. To address this gap, we conducted a realist systematic review of existing research evidence on immunization data use interventions in low- and middle-income countries (LMICs). Our review was designed to answer two specific research questions:

1. What are the most effective interventions to improve the use of data for immunization program and policy decision-making?
2. Why do these interventions produce the outcomes that they do?

## Methods

To answer our research questions, we conducted a realist review of the evidence from published and grey literature. Realist review is a theory-driven type of literature review that aims to test and refine the underlying assumptions for how an intervention is supposed to work and under what conditions [9, 10]. While traditional systematic review approaches follow a highly specified methodology with predetermined eligibility criteria to answer a specific research question, the realist review methodology (described elsewhere) involves an equally rigorous process for systematic synthesis of evidence but is more iterative and methodologically flexible [9, 10].

We developed a review protocol (Appendix A) with input from a technical steering committee composed of ten global and regional senior leaders in the areas of immunization, data quality, and data use from the Pan American Health Organization (PAHO), World Health Organization (WHO) headquarters, the Bill & Melinda Gates Foundation, PATH, the US Centers for Disease Control and Prevention, the United Nations Children’s Fund, and Gavi, as well as country representatives from both the Better Immunization Data Initiative (BID Initiative) Learning Network and Improving Data Quality for Immunizations core project countries.

**Fig. 1:**
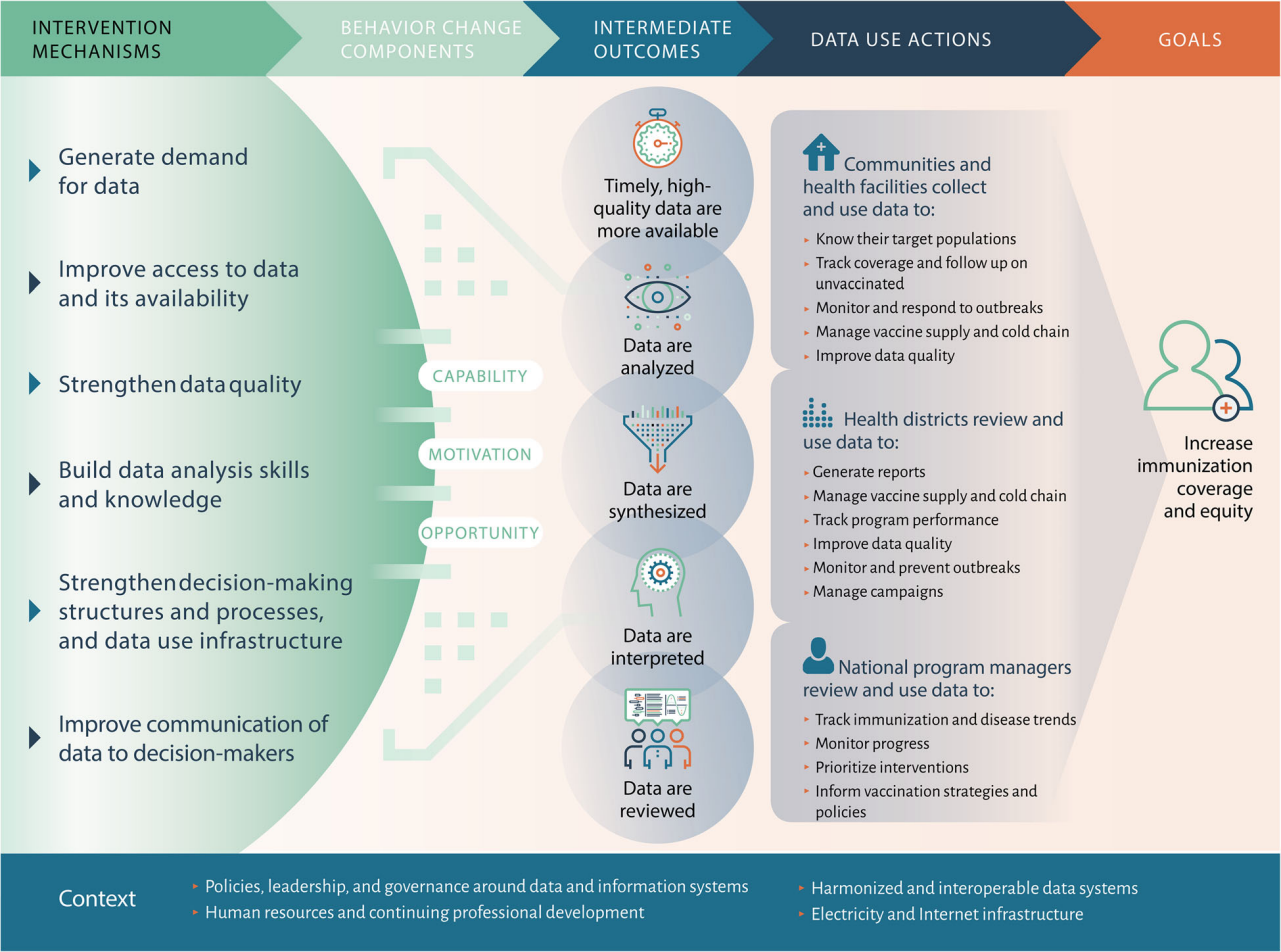
Theory of Change for Supporting Data-Informed Decision-Making for Immunization Programs

We first developed a theory of change (TOC) (Fig. 1) to establish the theoretical framework for how we expect data use interventions to influence data use. The hypotheses and assumptions reflected in the TOC were informed by existing health information and data use frameworks, as well as systematic reviews on topics related to health information system strengthening and evidence-informed decision-making [11–16]. We adopted the WHO definitions of data quality and data use. WHO’s data quality review framework defines data quality according to four dimensions: completeness and timeliness, internal consistency of reported data, external consistency, and external comparisons of population data [17]. Data-informed decision-making is defined by WHO as a process in which data collected by the health system are converted into usable information through data processing, analysis, synthesis, interpretation, review, and discussion, then used to decide on a course of action [18]. From this literature, we identified six barriers to data use (demand, access and availability, quality, skills, structure and process, and communication) and three behavioral drivers (capability, motivation, and opportunity), which are represented in the TOC as *mechanisms* of data use interventions [11–14]. We hypothesize that to be effective, any intervention must address one or more of these mechanisms. Likewise, we expect that interventions addressing these mechanisms will lead to *intermediate outcomes* including data quality and availability, analysis, synthesis, and discussion of data, which we posit are also necessary precursors to data use. From this point, the actual use of data to make program and health service delivery decisions is captured by the *data use actions*, which are based on the WHO *Global Framework to Strengthen Immunization and Surveillance Data for Decision-making* [15]. The data use actions represent our outcome of interest in this review; they specify where data are used, by whom, and for what purpose.

### Literature search

We searched PubMed, POPLINE, Centre for Agriculture and Biosciences International Global Health, and African Journals Online for published articles. Search terms included *vaccine*, *immunization*, *data quality*, *data use*, *health information system*, *health management information system*, *logistics management information system*, *electronic medical record*, *electronic health record*, *electronic patient record*, *medical record system*, *immunization register*, *home-based record*, and *supply chain data*. The search strategies retained for each database are included in Table 1. We purposively filled gaps with additional searches on specific intervention categories. We also performed “reference mining” by searching the references of retrieved articles for additional relevant publications. Using the same search terms, we searched for grey literature on vaccine and digital health conference, implementer, and technical agency websites. A complete list of the websites searched for grey literature is included in the review protocol (Appendix A). We collaborated with colleagues from PAHO to collect literature from country offices in the Latin America and Caribbean region and relied on input from the steering committee to identify immunization data use interventions and implementing organizations. We contacted these organizations to collect unpublished evaluations, studies, and reviews of data use interventions. The first round of searches was conducted between January and April 2018 and a second round was conducted between June and August 2018. During the second round, we included literature from other health sectors such as HIV/AIDS and maternal and child health to fill gaps in the immunization evidence.

**Table 1:** Search Strategies

Database	Search strategy
PubMed	((vaccin*[Title/Abstract] OR immunis*[Title/Abstract] OR immuniz*[Title/Abstract]) OR (immunization or immunisation or vaccine[MeSH Terms])) AND (“data quality“[Title/Abstract] OR “data use“[Title/Abstract] OR “data-use“[Title/Abstract])
POPLINE	((data use) OR (data-use) OR (data quality)) and (Keyword: vaccines OR Keyword: immunization)
CABI Global Health	(Abstract: (data use) OR (data-use) OR (data quality) OR title: (data use) OR (data\-use) OR (data quality)) AND (Abstract: (health management information system) OR (electronic medical record) OR (immunization register) OR (home\-based record) OR (logistic management information system) OR (supply chain data) OR (medical record system) OR (electronic health record) OR (electronic patient record) OR (health information system)) OR Title: ((health management information system) OR (electronic medical record) OR (immunization register) OR (home\-based record) OR (logistic management information system) OR (supply chain data) OR (medical record system) OR (electronic health record) OR (electronic patient record) OR (health information system)) AND (Abstract: (vaccin* or immuniz* or immunis*))
African Journals Online	((data use) OR (data-use) OR (data quality)) and (Keyword: vaccines OR Keyword: immunization)

### Inclusion criteria

During the first round of data collection, literature that met all the following criteria was eligible for inclusion:

1. **Focus is on routine health system data.** The literature reported on use of routine immunization data, which we defined as data that are continuously collected by health information systems and used by immunization programs to monitor and improve service delivery. This excluded surveillance data used for detecting disease outbreaks; financial and human resources data; and other nonroutine data, such as survey data and research evidence.
2. **An intervention is reported.** The literature reported on an intervention designed to improve routine data use.
3. **Data use outcomes are reported.** The literature reported on intervention outcomes related to data use for decision-making.
4. **Intervention targeted data users.** The intervention targeted health care professionals (e.g., health workers, managers, and decision-makers) as the principal users of routine data. This excluded interventions that targeted recipients of health care services (e.g., patients or communities).

The second round of data collection expanded the first criterion to include literature from other health sectors while all other criteria remained the same. We included systematic reviews that met our inclusion criteria during the second round of data collection and consulted the primary studies for additional information only if the results of the primary studies were not sufficiently detailed in the systematic reviews.

### Data extraction and study quality assessment

A six-step process was used in which we:

1. Read the literature abstracts to determine if they met the inclusion criteria.
2. Classified each piece of included literature based on the primary data use intervention type.
3. Read the full text of included articles and coded text segments using Atlas.Ti.
4. Extracted characteristics of the intervention package, including the intervention design and strategies, the types of health care professionals and levels of the health system targeted by the intervention, implementation settings, outcomes, and details on how the interventions functioned.
5. Assessed the quality of the immunization data use articles categorized as *evidence* using the Mixed Methods Appraisal Tool checklist [19].
6. Compiled the immunization data use metadata for each record in a Microsoft Excel workbook and visualized using Tableau in an evidence gap map.

Articles were read by a three-member review team and coded according to a coding tree based on the TOC. Approximately 20 % of the articles were cross-read and coded to ensure consistent coding among reviewers.

### Analysis

We did not exclude literature based on study design or quality, but rather segmented the included literature into two categories: *evidence* referred to studies and evaluations that applied scientific research methods or evaluation design, and *promising strategies* referred to grey or published literature that did not qualify as a study or evaluation but described an intervention with strong theoretical plausibility of improving data use.

For each intervention category, we analyzed the intervention’s effect on data use by health care professionals at different levels of the health system. We recorded how the interventions functioned and what mechanisms made them successful, as well as the reasons why interventions did not show evidence of effectiveness. We synthesized the results according to the intermediate outcomes of data quality and availability; data analysis, synthesis, interpretation, and review; and data use actions at different levels of the health system, as conveyed in our TOC. By mapping the evidence to the TOC, we tested the hypothesized relationships between intervention strategies, mechanisms, and data use outcomes and visualized the results in an evidence gap map. We presented a synthesis of our preliminary findings during a workshop held in Washington, DC, May 16 and 17, 2018, with members of the steering committee and other immunization stakeholders. During the workshop, we identified gaps in the immunization literature and decided to conduct a second round of data collection. For intervention categories that had limited evidence and were applicable outside of immunization, we expanded the review to include evidence from other health sectors.

### Assessing strength of evidence

For each intervention category, we rated the strength of evidence that the intervention resulted in the intermediate outcomes and data use actions outlined in our TOC. We assigned a strength of evidence rating of high, moderate, low, or very low based on a subjective estimation of four domains: (a) study design; (b) quality; (c) number of studies and their agreement; and (d) context dependence of the evidence (Table 2).

**Table 2:** Domains Assessed to Determine Strength of Evidence

Domain	Explanation
Study design	We considered experimental and quasi-experimental designs to improve the strength of estimates of intervention effectiveness. We considered experimental designs to provide the highest-strength evidence. However, other methods may be more important for assessing strength of claims on how and why the intervention works.
Quality	We used the Mixed Methods Appraisal Tool checklist to score the quality of the literature on routine immunization data use that qualified as evidence. ‘Strong’-quality studies scored 75–100 %; ‘Moderate’-quality studies scored 50–74 %; ‘Weak’-quality studies scored 0–49 %.
Number of studies and their agreement	A greater number of studies with similar findings improved our certainty in those findings. Studies with conflicting findings weakened the strength of evidence.
Context dependence	We considered evidentiary claims for highly context-dependent interventions to have lower strength, or we specified the conditions under which the claims hold true. For example, for certain interventions composed of multiple strategies, it was not possible to fully disentangle the effects of individual strategies. In such cases, we recognized how other strategies may have influenced the overall effect of the intervention.

## Results

Search results.

**Fig. 2:**
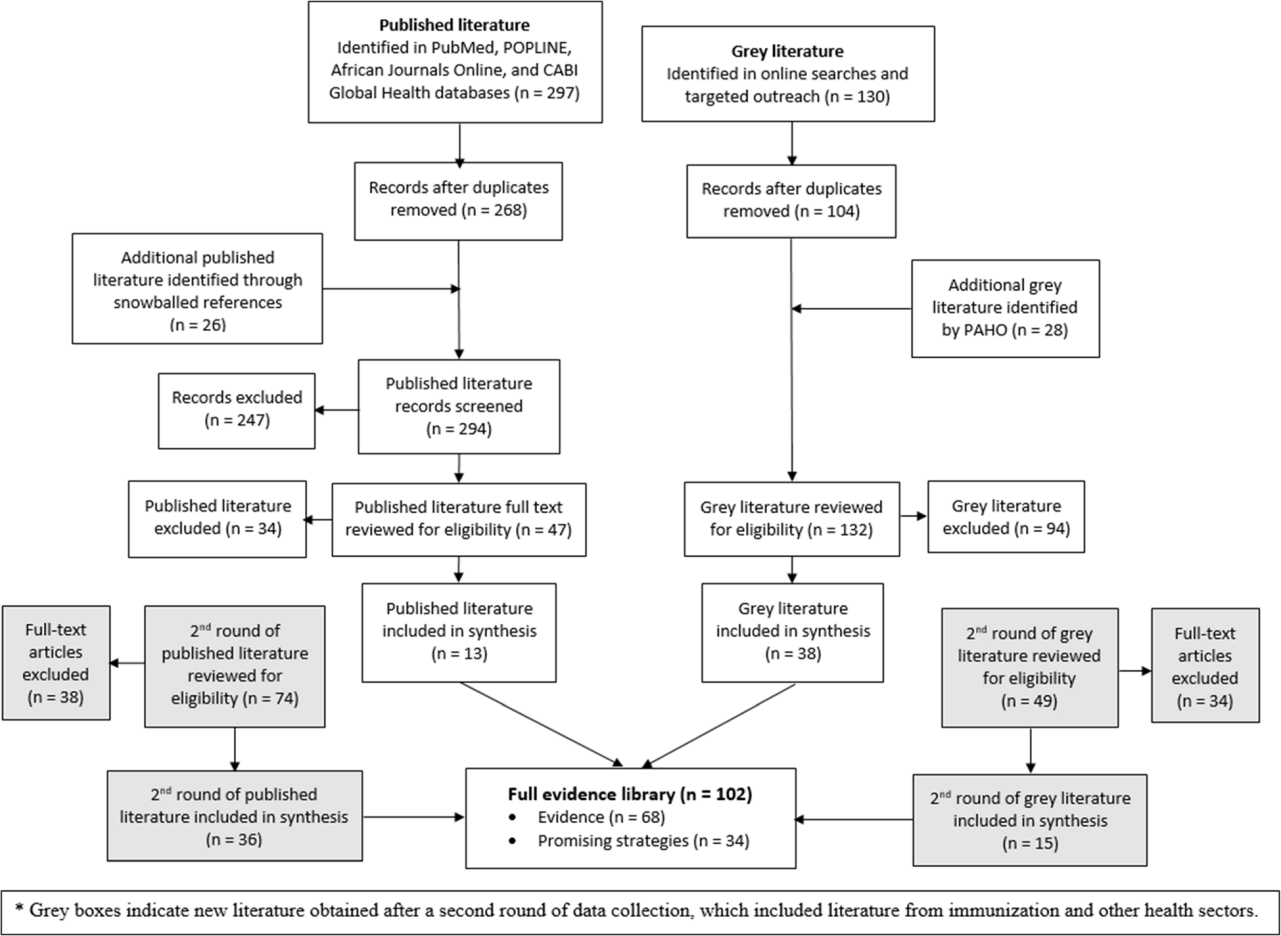
Literature Flow. Abbreviations: CABI, Centre for Agriculture and Biosciences International; PAHO, Pan American Health Organization.

The database searches yielded a total of 426 articles in the first round and an additional 123 articles in the second round of data collection (Fig. 2). Of these 549 articles, 102 met our inclusion criteria, including 49 from peer-reviewed journals and 53 from grey literature. A total of 66 articles reported on immunization data use interventions and 36 articles reported on data use interventions for other health sectors. We categorized 68 articles as research *evidence* and 34 articles as *promising strategies*. Ninety-five articles concerned interventions implemented in LMICs and seven articles related to interventions in high-income countries. We identified ten primary intervention categories (Table 3). Electronic immunization registries (EIRs) were the most reported primary intervention (*n* = 14), followed by decision support systems (*n* = 13) and multicomponent interventions (*n* = 13). Most articles described a primary intervention type and were often complemented by secondary intervention components. For example, EIRs were typically implemented with training and/or supportive supervision. We categorized an intervention as multicomponent when there were multiple equally emphasized strategies.

**Table 3:** Data Use Intervention Categories

Intervention category	Articles on immunization data use (n)	Articles on data use in other health sectors (n)	Total number of articles (n)	Description	References from peer-reviewed literature	References from grey literature
Electronic immunization registries	14	0	14	Store data on administered vaccine doses in computerized, population-based databases	[20–24]	[25–33]
Logistics management information systems	8	0	8	Collect data on vaccine inventory and demand to support managing the vaccine supply chain; often computerized	[34]	[35–41]
Health management information systems	0	6	6	Store aggregated health data and can facilitate converting data into useful information for decision-making; we focused on computerized HMIS	[1, 42–46]	
Decision support systems	9	4	13	Help users interpret data and use data for decision-making; include computerized decision support systems and noncomputerized tools (e.g., monitoring charts, dashboards, and home-based records)	[47–53]	[54–59]
Data quality assessments	8	3	12	Range from interventions that train program managers in how to routinely audit data quality to external audits of data quality	[60–65]	[66–71]
Data review meetings	2	1	3	Employ adult learning techniques (e.g., peer learning and knowledge-sharing) to build skills in data analysis	[72, 73]	[74]
Peer learning networks	6	5	11	Connect health workers so they can share information and discuss data; increasingly accessed through social networking platforms online	[75–77]	[78–85]
Supportive supervision, mentorship, and on-the-job training	4	4	8	Build health workers’ skills, foster performance and motivation, and identify and resolve problems	[86–91]	[92, 93]
Training	1	5	6	Strengthen the capacity of health workers responsible for managing and using data at all levels of the health system through workshops, classroom-based learning, and hands-on approaches	[94–97]	[98, 99]
mHealth	7	1	8	Mobile-based or wireless technologies used to support health service delivery	[100–103]	[104–107]
Other/multicomponent interventions	7	6	13	Leverage many of the intervention categories but lack a clearly identifiable primary intervention type	[2, 108–111]	[112–119]
**TOTAL**^a^	**66**	**36**	**102**			

^a^A total of 102 articles were included in the review, including 49 articles from peer-reviewed literature and 53 articles from grey literature.

**Fig. 3:**
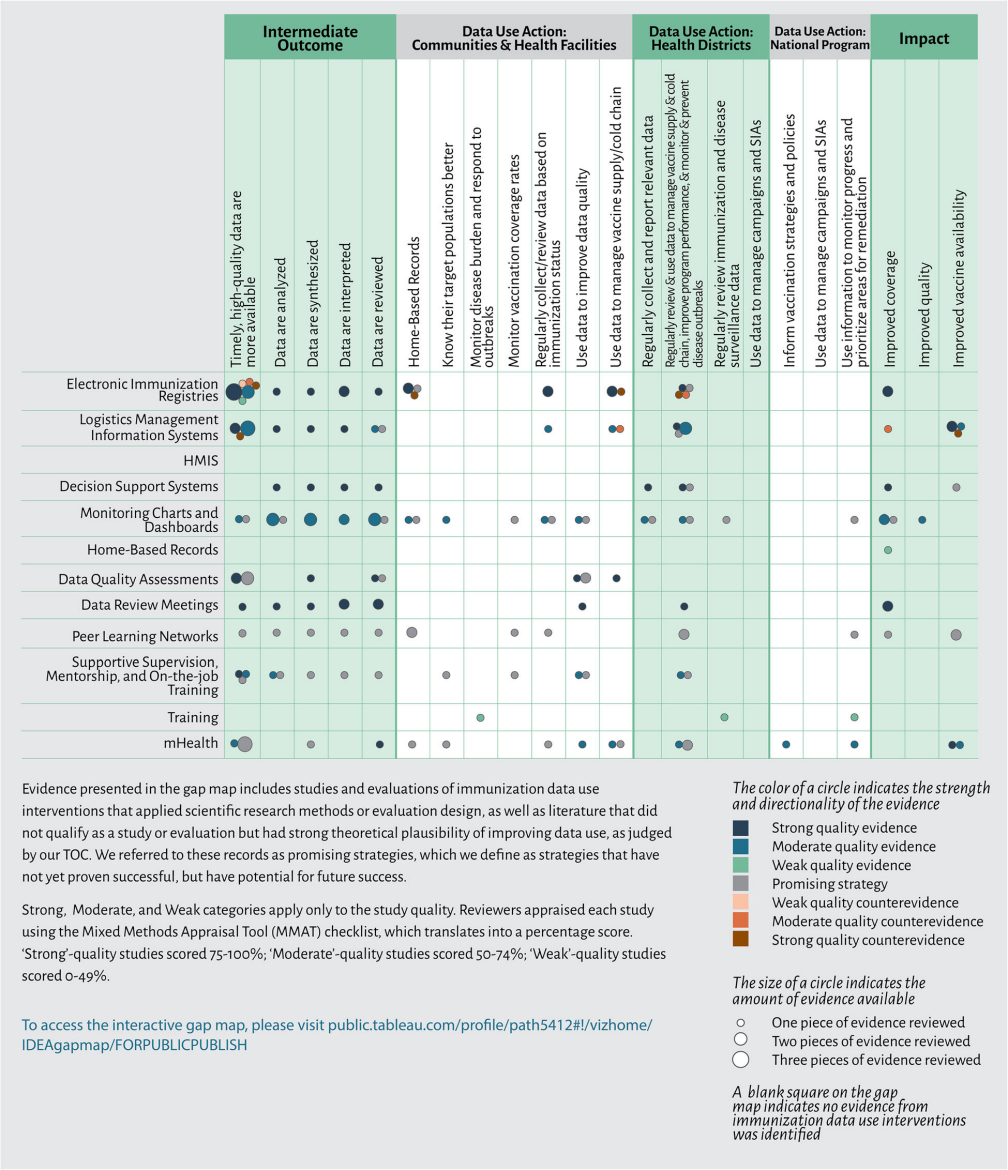
Evidence Gap Map. Abbreviations: HMIS, health management information systems; mHealth, mobile health; SIA, supplementary immunization activity.

We plotted the results of the 66 articles that reported on immunization data use interventions by primary intervention type and evidence of intermediate outcomes and data use actions in an evidence gap map (Fig. 3). The gap map visualizes all pieces of evidence, including the strength and directionality of the evidence, and promising strategies. A complete list of the immunization data use articles and their quality appraisal scores is included in Appendix B. A detailed synthesis of evidence by intervention category and data use outcomes is included in Appendix C.

**Table 4:** Summary of Included Articles that Reported on Intermediate Outcomes and Data Use Actions

	Articles reporting on immunization data use interventions		Articles reporting on data use interventions in other health sectors		Sub-total		
	Evidence (*n* = 37)	Promising strategies *(n* = 29)	Evidence (*n* = 31)	Promising strategies (*n* = 5)	Evidence (*n* = 68)	Promising strategies (*n* = 34)	Total (*n* = 102)
**Intermediate outcomes**							
Availability of timely, high-quality data	27 (72 %)	15 (52 %)	8 (26 %)	0 (0 %)	35 (51 %)	15 (44 %)	50 (49 %)
Data are analyzed, synthesized, interpreted, and reviewed	15 (41 %)	11 (38 %)	8 (26 %)	0 (0 %)	23 (34 %)	11 (32 %)	34 (33 %)
**Data use actions**							
Data use in communities and health facilities	13 (35 %)	16 (55 %)	8 (26 %)	0 (%)	21 (31 %)	16 (47 %)	37 (36 %)
Data use at the district level	12 (32 %)	12 (41 %)	5 (16 %)	1 (20 %)	17 (25 %)	13 (38 %)	30 (29 %)
Data use at the national level	2 (5 %)	5 (17 %)	1 (3 %)	0 (0 %)	3 (4 %)	5 (15 %)	8 (8 %)

### Overview of included articles

#### Intermediate outcomes: availability of timely, high-quality data

A total of 35 articles reported evidence related to data availability, timeliness, and/or quality (Table 4). Among the immunization literature, 22 articles reported an improvement in the availability, timeliness, and/or quality of data. Digital information system interventions (EIR, logistics management information systems [LMIS], and HMIS) were the most common intervention type to report improvements in data availability [20–22, 25] and data quality [1, 20, 22, 25–27, 42, 120]. Five articles, mostly EIR interventions, did not find any effect on data availability, timeliness, and/or quality [24, 26, 28, 29, 35]. There was moderate-strength evidence that Digital information system interventions were associated with improved data quality owing to the ease of automated feedback and functionalities such as logical checks and warning prompts for improbable or missing data entries [121]. In the included literature from other health sectors, a systematic review of District Health Information Software 2 (DHIS2) implementation found that as health workers gained timelier access to data through the electronic platform, they reported a greater sense of ownership and responsibility for producing high-quality data [42]. Likewise, a multi-country case study of DHIS2 in seven African countries found that more data use generated demand for higher-quality data [1]. Digital information system interventions that were reinforced by other data use activities were more likely to improve data quality than interventions that did not account for structural, behavioral, and other barriers. For example, a nonexperimental, mixed-methods study in South Africa found that human resources shortages, limited awareness of the importance of data, and lack of support and feedback mechanisms undermined the effectiveness of the DHIS platform at improving data quality [43]. Challenges at the point of data entry into digital information systems also lessened data quality, especially data completeness. Numerous articles reported inconsistent data entry due to heavy workloads, parallel paper and electronic reporting systems, disruption to existing workflows, unreliable internet connection, lack of trained staff, and faulty equipment [22, 26, 28, 54]. There was low-strength evidence to suggest that challenges associated with manual data entry could be overcome by digitization of paper immunization records and other mHealth solutions [22, 23, 26, 30–32, 100]. 

There was moderate- to high-strength evidence of improved data quality from repeated data quality assessments and low-strength evidence for data review meetings as part of broader efforts to develop health information infrastructure [60–63, 66–68, 72]. These interventions worked by bringing greater visibility and awareness to data quality issues. Likewise, interventions were more effective when implemented alongside supportive supervision and other forms of feedback that held health workers accountable and developed their skills to address data quality issues [60, 64, 72]. 

#### Intermediate outcomes: data are analyzed, synthesized, interpreted, and reviewed

A total of 23 articles reported evidence related to data analysis, synthesis, interpretation, and review. Among the immunization literature, 14 articles reported an improvement in outcomes related to data analysis and interpretation, which we considered necessary steps for transforming data into useful information for decision-making. Moderate-strength evidence suggested that health workers using digital information systems had increased ability to synthesize and interpret routine data, such as identifying defaulters, areas of low coverage, and vaccine stock levels [23, 25, 26, 47]. This outcome was more commonly reported at district and provincial than facility levels. One nonexperimental mixed-methods study found no evidence of improvements in data analysis and interpretation by health facility workers, which was attributed to the absence of feedback and support mechanisms [24]. Instead, interventions that supported health workers in making sense of their data, such as data review meetings, monitoring charts and dashboards, and training, were most likely to report improvements in data analysis and interpretation [48, 49, 55, 62, 94]. There was moderate-strength evidence that monitoring charts and data dashboards, both paper and electronic, increased tracking of immunization coverage [48, 54, 55]. These tools helped health workers synthesize disparate pieces of data, thus improving their ability to detect and react to problems. They were most effective when integrated within established data review and decision-making processes, such as monthly review meetings, and reinforced by supportive supervision and other forms of feedback. The content of data review meetings and how they were structured also influenced their success. For example, building on recommendations and discussion from previous meetings reinforced learning [73], while emphasizing data completeness and accuracy over immunization program target achievement may have improved attitudes about data use [78, 122]. More effective were data review interventions that incorporated quality improvement approaches, such as Rapid Appraisal of Program Implementation in Districts and Plan-Do-Study-Act cycles, because they provided a structured approach to problem-solving [73]. Low-strength evidence suggests that peer learning networks increased collaborative data review and problem-solving by health workers [78, 79, 123]. These interventions leveraged social network platforms like WhatsApp and other forums to bring together health workers across health system levels, departments, and functions, which helped motivate health workers and build their analytic skills. 

#### Data use actions: data use in communities and health facilities

A total of 21 articles reported evidence related to data use at the community and health facility levels. Among the immunization literature, 11 articles reported an improvement in data use by frontline health workers and two articles found no evidence of improvement. At the facility level, data use interventions placed more emphasis on improving data collection practices and data quality. There was low-strength evidence that health facilities used data from digital information systems to make decisions and take action. Two nonexperimental mixed-methods studies of EIR interventions found an increase in facility health workers who self-reported using data to guide their actions [25, 27], but other studies of EIR and HMIS interventions did not detect a change [26, 29, 43, 44]. Challenges such as data entry burdens, poor infrastructure, and lack of feedback from the district and higher levels contributed to inconsistent use. 

There was low- to moderate-strength evidence from other interventions. Three articles found that health facilities used monitoring charts and data dashboards to review whether they were meeting targets, respond to low vaccine coverage, and follow up on defaulters [49, 54, 55]. Data quality assessments were the most common intervention type to result in data use by health facilities but centered on resolving data quality issues rather than improving service delivery [60–62, 66–68]. Supportive supervision interventions targeting the facility level, such as the Data Improvement Team intervention in Uganda, showed mixed evidence of effectiveness. Results from routine project monitoring found an increase in the proportion of health facilities with documented evidence that routine immunization data were used for decision-making, but a rapid organizational-level survey found that none of the health facilities sampled had implemented Data Improvement Team recommendations related to data use [86, 92]. Reasons for inaction included insufficient availability of required materials, inadequate human resources capacity (e.g., new and untrained staff, and low motivation), and a poor management structure that lacked clarity around roles and responsibilities related to data analysis and use. Training in various forms and intensities was a secondary component in at least 17 interventions reviewed. There was low-strength, mostly anecdotal, evidence that one-off training interventions contributed to data use [95, 124]. However, when training was implemented as part of a multicomponent intervention, or reinforced by supportive supervision, these interventions had moderate-strength evidence of improving data use [96–98, 108]

#### Data use actions: data use at the district level

A total of 17 articles reported evidence related to data use at the district level. Among the immunization literature, ten articles measured an improvement in data use by health districts and two articles found no change. LMIS interventions were the most common intervention type to find evidence of increased data use by health districts. Reported data use outcomes were related to supply chain management and included data use in vaccine forecasting and delivery, response to stockouts and cold chain equipment breakdowns, and decisions involving monitoring and supervising health facilities [23, 36, 100, 109]. LMIS interventions that leveraged additional data use strategies were most effective. In Mozambique, for example, the success of the Dedicated Logistics System was influenced by recruiting logisticians responsible for data collection and entry, thus relieving facility health workers from the task; incorporating built-in data visualization to support analysis; and coordinating monthly data review meetings to identify bottlenecks and solutions to improve performance [125]. 

Similar to LMIS, EIR interventions assume that making data more available and accessible to users will lead to improvements in data use. Although EIRs were the most common data use intervention in our review, only four studies and evaluations measured data use outcomes. Project data from EIR interventions in Tanzania and Zambia found an increase at project midline in the proportion of district-level health workers who self-reported taking action in response to their data [25, 27], although an external evaluation of the same intervention in Tanzania found no significant change in data use between baseline and midline [26]. The evaluators noted that it may have been too early to measure significant changes because of multiple implementation delays. An evaluation of the SmartCare electronic medical record intervention in Zambia found no effect on data use, since most facilities were not entering immunization data into the system; in addition to challenges with the acceptability and feasibility of the system, health workers could not identify ways to use data for action [29]. Other studies of EIR interventions did not measure data use outcomes but found improvements in vaccine coverage that could have been associated with data use if health workers used EIR data to follow up on defaulters and target under-immunized children [20, 21]. 

Decision support system interventions, such as monitoring charts and data dashboards, helped district health workers organize and analyze data and then use the information to strengthen facility performance and data quality. Project data from the implementation of an immunization data dashboard in Nigeria’s DHIS2 platform found that at state and local government area levels, health workers used the dashboard to track facility performance, monitor immunization coverage trends, and target facilities for training or supportive supervision [54]. Effectiveness was enhanced by deploying DHIS2 implementation officers to provide hands-on learning and support to state and local government area immunization teams, and incorporating data use within existing processes, such as monthly review meetings. In these literature and other studies, decision support systems had moderate-strength effectiveness [73, 74, 92]. Conversely, computerized decision support systems (CDSS) had low-strength evidence of effectiveness. Such systems employ algorithm-based software to help data users interpret and transform data into usable information for decision-making. A mixed-methods evaluation of a CDSS intervention in Papua New Guinea found that district health workers in low-performing regions were more likely to use the knowledge-based system to give feedback to health facilities, which gave rise to the immunization rate, but health workers from higher-performing districts did not perceive any utility in reviewing data [47]. The literature on CDSS from other health sectors and settings did not show an effect on data use or clinical outcomes [50, 51].

#### Data use actions: data use at the national level

A total of three articles, including two from the immunization literature and one from the other health sector literature, reported evidence related to data use at the national level. Many data use interventions for national-level stakeholders did not meet our inclusion criteria because they focused on decision-making informed by research evidence and survey data instead of routine data. The literature we found provided low-strength, often anecdotal, evidence of data use. For example, in Ghana and Kenya, anecdotal evidence suggested that immunization information system assessments led to concrete follow-up actions, such as improving the managerial and supervisory skills of subdistrict staff in Ghana and incorporating data quality into coursework and continuing education curricula for health professionals [65]. Peer learning networks such as the BID Initiative Learning Network, found that Expanded Programme on Immunization managers and other national-level participants self-reported becoming more data oriented in their work and making decisions based on data [27]. The Data for Decision making project, which included interdisciplinary in-service training for mid-level policymakers, program managers, and technical advisors, found anecdotal evidence of data use for strengthening surveillance systems, and for advocating for, developing, and implementing national policies [95].

## Discussion

We found that the state of the evidence around what works to improve immunization data use is still nascent. Although much of the published literature provides insights into the barriers related to data use [4–8], few data use interventions have been rigorously studied or evaluated. We found more evidence of interventions impacting the intermediate outcomes in our TOC, such as data quality, availability, analysis, synthesis, interpretation, and review, but less evidence on what works to support data-informed decision-making. This could be explained by the lack of consensus around how to define and measure data use. Although *promising strategies* have not yet proven effective, we included them because they provide insight into intervention designs that have potential for future success

By applying a realist review methodology, we developed stronger theories about what works to improve the use of data based on the evidence and promising strategies currently available. We concluded that multicomponent interventions with mutually reinforcing strategies to address barriers at various stages of data use were most effective. Furthermore, interventions were more likely to succeed and be sustained over the long term if they institutionalized data use through dedicated staff positions for data management, routine data review meetings, national training curricula, and guidelines on data use for frontline staff. We found potential for digital systems but note that barriers still exist. While the transition from paper to digital, along with adoption of digital information systems, has made higher-quality data more available to decision-makers in real-time, it has not automatically translated into greater data use. There is more success at the district level and higher because of fewer operational challenges than at the facility level. It is also necessary to pair digital systems with activities that reinforce data use.

On the topic of data quality, the results of this review confirm that data quality is an important barrier and necessary precursor to data use, but we found limited evidence that interventions focused singularly on data quality improved data use. This is because health workers may lack the necessary skills to analyze and translate data into information that is useful for making decisions on program implementation. There is more compelling evidence to suggest that data use interventions can lead to improvements in data quality. We found that as health workers began using their data, they were able to identify inconsistencies and take corrective action. Data use also generated demand for higher-quality data, and as quality improved, users better trusted the data that reinforced use of the data.

Our primary focus was on the use of immunization data in LMICs, but we included evidence from other health sectors and relevant publications from high-income countries that further corroborated and deepened our findings. We found considerable evidence on improving the quality and use of HIV data, owing in large part to the strategic focus and investments in data use by PEPFAR. Our conclusions agree with other literature on the topic. For example, the finding that multicomponent interventions are likely more effective than single-component interventions is supported by other health systems research [6, 108].

We noted particular gaps in the evidence on what works to improve data use at the facility level. Our findings suggest that interventions at this level have focused more on improving data collection practices and data quality, and less on data use. More emphasis on building data use skills during frontline health worker pre- and in-service training and continuing education (e.g., statistics, data interpretation, and data management) and cultivating a culture of data use may have a greater effect on strengthening data quality and use, but this should be tested in future research. In addition, the operational barriers and administrative challenges faced by digital information system interventions point to the need for a phased approach, ensuring that data use infrastructure, human resources capacity, and skills-building are in place before a full digital transition.

We posit that the lack of consensus around how to define and measure data use may in part explain the dearth of rigorously evaluated data use interventions in the published and grey literature. There is therefore a critical need to develop better measures for assessing data use in decision-making to better understand the effectiveness of these interventions. Evaluation designs must also account for complex interventions, which encompass most of the interventions reported here. We do not necessarily recommend investment only in experimental design studies to establish effectiveness; rather, we found that the most useful and richest evidence came from mixed-methods studies and evaluations that described why and how the intervention worked, for whom, and where it worked.

### Strengths and limitations

A key strength of this review was its inclusiveness and methodological flexibility, afforded by the realist review approach. Realist review methodologies are increasingly used for synthesizing evidence on complex interventions because of their suitability for examining not only whether an intervention works, but also how the intervention works and under what conditions. The realist approach made it possible to include various types of information and evidence, such as experimental and nonexperimental study designs, grey literature, project evaluations, and reports. Although much of the evidence was from grey literature and therefore of lesser quality, it provided important evidence and learnings that otherwise would be overlooked. In addition to being the first systematic synthesis of evidence on data use interventions in LMICs, our review adds to the growing body of realist review literature by demonstrating realist methodology applied to the review and synthesis of evidence in public health.

Most data use interventions were composed of multiple strategies, and although we attempted to segment the findings according to the primary intervention type, it was not possible to fully disentangle the effects of individual strategies and activities. For this reason, we cannot recommend which interventions or packages of interventions are most effective, but we can provide stronger theories about what may work and why. Another limitation was our reliance on what was reported in the literature that provided the basis for our findings. Not all the literature adequately described how the intervention functioned or identified the contextual factors within which the intervention was implemented that may have contributed to its success or failure. Because we did not have the opportunity to interview the stakeholders responsible for implementing the interventions, we may have missed important contextual considerations.

Finally, the focus on routine immunization data alone was helpful in constraining the review timeline and process but risks further siloing of immunization programs. We eventually expanded the review to include literature from other health sectors; however, these efforts were not as comprehensive and likely failed to capture all the available evidence on the topic. By including systematic reviews during the second round of data collection, we were able to capture evidence from data use interventions that have already been synthesized for other health sectors. The results of this review, along with other future reviews of data use more broadly, should be considered together to inform strategic and cross-programmatic investments in interventions to improve data use.

## Conclusions

This review helps fill a critical gap in what is known about the state of the evidence on interventions to improve routine health data. While our findings are presented primarily through the lens of using data to make decisions in immunization programs, they remain relevant for other health sectors. The evidence on effective interventions and promising strategies detailed in this review will help program implementers, policymakers, and funders choose approaches with the highest potential for improving vaccine coverage and equity. We anticipate that these findings will also be of interest to researchers and evaluators to prioritize gaps in the existing knowledge.

## Supplementary Information

**Additional file 1:**

**Additional file 2:**

**Additional file 3:**

## Data Availability

The article names of published and grey literature that support the findings of this study are available in the interactive evidence gap map (linked here: https://www.technet-21.org/en/topics/idea#evidence-gap-map). Complete citations for all referenced literature are included in the reference list.

## Abbreviations

AIDS: Acquired Immune Deficiency Syndrome
BID Initiative: Better Immunization Data Initiative
CABI: Centre for Agriculture and Biosciences International
CDSS: Computerized decision support system(s)
DHIS2: District Health Information Software 2
EIR: Electronic immunization registry
Gavi: Gavi, the Vaccine Alliance
HIV: Human immunodeficiency virus
HMIS: Health management information system(s)
LMICs: Low- and middle-income countries
LMIS: Logistics management information system(s)
mHealth: Mobile health
PAHO: Pan American Health Organization
PEPFAR: US President‘s Emergency Plan for AIDS Relief
POPLINE: Population Information Online
TOC: Theory of change
WHO: World Health Organization
