# Supplementary material for: A realist systematic review of evidence from low- and middle-income countries of interventions to improve immunization data use
Source: BMC Health Serv Res. 2021 Jul 8;21:672. doi: 10.1186/s12913-021-06633-8 (PMC8268169; doi:10.1186/s12913-021-06633-8)
Supplement: Supplementary file 1 — Additional file 1: [file 12913_2021_6633_MOESM1_ESM.docx]

Appendix A. Review protocol

**Background**

Over the past several years, there has been an increase in efforts to address the challenges countries face with their immunization data quality and use for making decisions around health service delivery. However, there are no concise mechanisms in place to share the best practices and knowledge generated across these efforts and help inform how new initiatives can effectively and efficiently improve immunization data use. Furthermore, we are not aware of any formal review of evidence from existing efforts to strengthen immunization data use. This study seeks to address this problem by identifying and synthesizing existing evidence from the published and unpublished literature.

**Methods**

We will take a realist review approach [1, 2] to answer our primary research questions:

1. What are the most effective interventions to improve the use of data for immunization program decision-making?
2. Why do these interventions produce the outcomes that they do?

Due to the nature of the research questions and the topic, and in line with other qualitative or mixed-methods review approaches [3], we are flexible in terms of the types of evidence that we will include as well as the quality of the evidence. We plan to include quantitative, qualitative, mixed-methods, and evaluation studies, as well as project reports. Instead of maintaining strict quality criteria, as in a traditional systematic review, we will prioritize evidence that answers our research questions and tests our TOC. To achieve this review’s objectives, we propose the six steps outlined in detail below.

**Step 1: Reviewing related systematic reviews and refining the theory of change**

This review will be guided by our proposed TOC (Figure 1). The TOC builds on existing health information and data use frameworks and logic models as well as systematic reviews on topics related to health information system strengthening and evidence-informed decision-making [4–9] to explain the hypothesized mechanisms and contextual factors underlying immunization data use. It draws from the context-mechanism-outcome (C-M-O) configuration suggested by Pawson [1] and Robert et al. [2] for studying complex social interventions. We propose outcomes that are framed as “data use actions,” which are similar to those proposed in the WHO *Global Framework to Strengthen Immunization and Surveillance Data for Decision-making* [8] and specify where data are used, by whom, and for what purpose.

**Fig. 1 Theory of Change for Immunization Data Use**


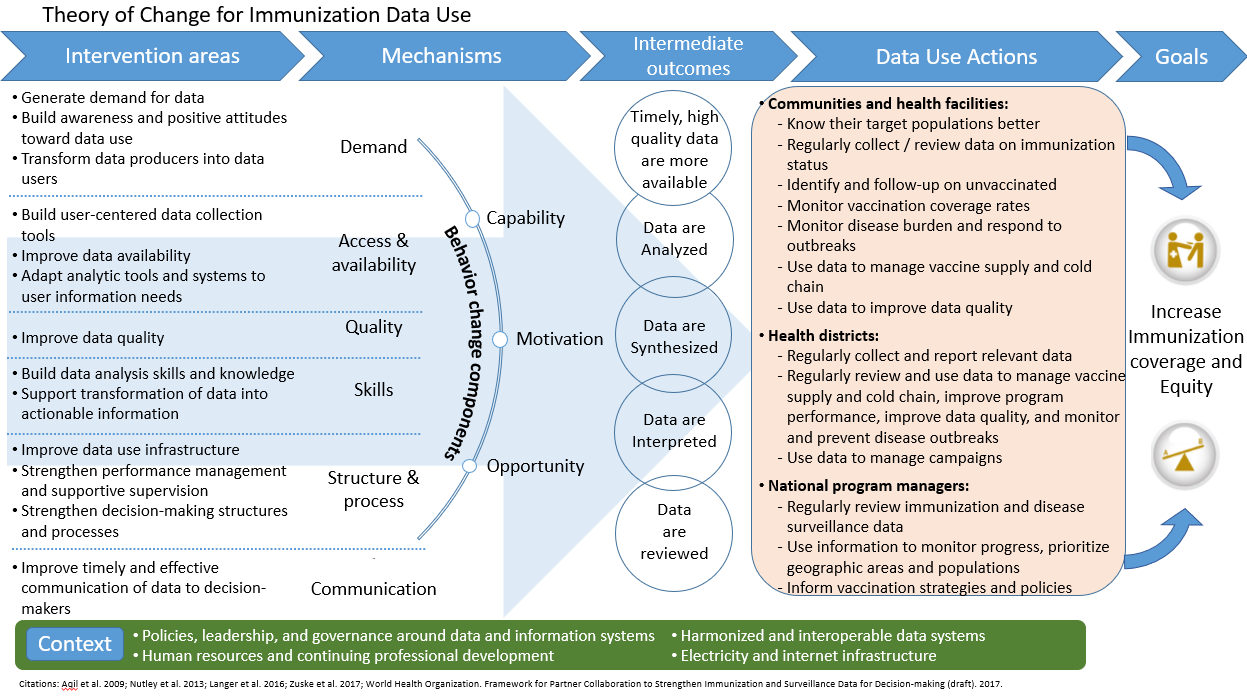


As was proposed by Langer et al. [6] in their logic model explaining how interventions lead to evidence-informed decision-making, we include behavioral mechanisms as intermediary mechanisms that relate to the individual-level drivers underlying how data are used for problem-solving and that mediate the intended outcomes in our framework. We assume even with the most appropriate data systems and data available, “use” is ultimately a human behavior. The proposed mechanism categories are influenced by Aqil et al.’s PRISM framework and Langer et al.’s *Science of Using Science* intervention logic model [5–6]. Table 1 defines each mechanism. We grouped intervention components from Langer et al. [6], Aqil et al. [5], and Nutley and Reynolds [4] by mechanism category. We proposed contextual factors based on the framework on health information use systems from the Swiss Tropical and Public Health Institute [7].

**Table 1 Mechanisms**

|  | **Definition** | **Reference** |
| --- | --- | --- |
| ***Mechanisms*** | | |
| Demand | Building demand for and positive attitudes toward data- and information-informed decision-making.  This mechanism emphasizes the importance of health workers, managers, and decision-makers valuing the concept of data-informed decision-making as well as the intermediate steps needed to achieve that goal. | [6] |
| Access & availability | Ensuring availability of data and then ensuring that potential users are able to access data.  This mechanism emphasizes our assumption that data must be available and accessible if the data are to be used. | [4,6] |
| Quality | Ensuring that data are of appropriate quality for the decisions or actions to be informed.  This mechanism emphasizes the fact that poor-quality data is often cited as a barrier to data use and that improvements in data quality may lead to improved data use. | [4] |
| Skills | Ensuring data users have the skills to access data and to turn data into actionable information through data management, analysis, synthesis, interpretation, and discussion. Users must be able to apply these skills to the workplace. Health workers, managers, and decision-makers should be able to integrate data and information with other drivers of decision-making. | [4,6] |
| Structure & process | Influencing organizational, technological, and institutional structures and processes that facilitate or block data-informed decision-making.  This could include how data management infrastructure is structured; how health workers spend their time and what latitude they have to take action; or professional norms related to collecting, analyzing, and discussing data. | [4,6] |
| Communication | Influencing the timely and effective communication of data to potential users or those in a position to take action.  Unlike “Access & availability,” this mechanism acknowledges that some decisions or actions will be based on data and information that are “pushed” to potential users and that simply making data and information available and accessible is often not sufficient. | [4,6] |
| ***Behavior change components*** | | |
| Capability | *Capability is defined as the individual's psychological and physical capacity to engage in the activity concerned. It includes having the necessary knowledge and skills.* | [11] |
| Motivation | *Motivation is defined as all those brain processes that energize and direct behaviour, not just goals and conscious decision-making. It includes habitual processes, emotional responding, as well as analytical decision-making.* |  |
| Opportunity | *Opportunity is defined as all the factors that lie outside the individual that make the behaviour possible or prompt it.* |  |

We will iterate and refine the TOC based on the results of the literature review to integrate new explanatory elements as they are identified. We will also seek input from the steering committee.

**Step 2: Systematic review of effectiveness (peer-reviewed literature)**

To help answer research question 1 (“What are the most effective interventions to improve the use of data for immunization program decision-making?”), we will employ a traditional systematic review strategy for identifying and reviewing primary studies. Our literature search strategy is designed to retrieve both relevant peer-reviewed/published and grey/unpublished/non-peer-reviewed documents, although these are broken into two steps for pragmatic purposes. Step 2 focuses on peer-reviewed literature, including experimental and observational studies, evaluations, and qualitative research cited in PubMed, POPLINE, Centre for Agriculture and Biosciences International Global Health, and African Journals Online.

We will search these databases using combinations of keywords to identify relevant documents (Table 2). The titles and abstracts identified in the searches will be exported and cataloged in a Microsoft Excel spreadsheet and will be reviewed for inclusion or exclusion by a team of two reviewers based on the criteria outlined in Table 3. These reviewers will perform a double review on randomly selected documents to minimize exclusion biases. Included documents will then be identified for quality assessment (Step 4) and data extraction (Step 5).

**Table 2 List of Keywords for the Document Search Strategy**

| **- OR -** | **- AND -** | **- OR -** | **Themes and phrases** |
| --- | --- | --- | --- |
| Vaccin*  Immunis*  Immuniz* | Data quality  Data use  Data-use | Health management information system  Health information system  Electronic medical record  Immunization register  Home-based record  Logistics management information system  Supply chain data  Medical record system  Electronic health record  Electronic patient record | Routine immunization program strengthening  Immunization OR vaccine data use  Expanded Programme on Immunization data  Immunization information  Data for action  Data-informed decision-making  Performance improvement |

“*” retrieves records that contain the search term and all possible suffix variations of a root word.

**Table 3 Inclusion and Exclusion Criteria**

| **Inclusion criteria** | **Exclusion criteria** |
| --- | --- |
| Focus on routine health system data (e.g., HMIS, EMR, immunization registers [paper and electronic], immunization cards, supply chain data/LMIS) | Focus on the use of research evidence, surveillance data, survey data, or other nonroutine sources and types of data |
| Focus on immunization data | Focus on other health sector data (e.g., MNCHN, reproductive health, HIV/AIDS, etc.) |
| Studies, evaluations, reports, and/or descriptions of interventions to improve routine data use (including data quality as an intervention) | Not specific to a particular intervention (e.g., studies describing the barriers or facilitators of data use) |
| Outcome examined is use of routine data for immunization decision-making | Outcome examined is data quality alone or something other than data use |
| Intended user of data is a health worker, decision-maker, or manager | Intended user of data is a patient or community |

Abbreviations: EMR, electronic medical record; HMIS, health management information system; LMIS, logistics management information system; MNCHN, maternal, newborn, and child health and nutrition.

As we review documents for inclusion or exclusion in relation to our two research questions, we will flag documents that discuss the facilitators or barriers of data use. While not included to answer our effectiveness question, we will examine these documents for additional data use mechanisms and refine the TOC accordingly.

References from the included documents will be reviewed using the “snowballing” technique to identify additional relevant documents, including grey literature.

**Step 3: Grey literature and program results**

The grey literature search will follow many of the same steps outlined for the peer-reviewed literature search described in Step 2 above. We will search the databases listed in Table 4 for evaluation reports, program reports, and policy documents from various sources. We will use our networks as well as the networks of the steering committee members to identify additional programs, projects, interventions, or activities that have aimed to improve immunization data use—whether or not they have been evaluated. We will aim to include examples of what has not worked to further test and strengthen our TOC.

**Table 4 Grey Document Sources**

| Grey literature | - Websites of targeted international organizations, nongovernmental organizations, and consulting firms, including:   - <https://www.jsi.com>   - <https://www.measureevaluation.org>   - <https://www.go2itech.org/>   - <https://www.villagereach.org/>   - <https://shifo.org/en/>   - <https://www.path.org>   - <https://www.msh.org/> - Vaccine and digital health conferences and webinars, including:   - <https://gdhf2020.dryfta.com>   - <https://bidinitiative.org>   - <https://www.technet-21.org/en/network/events> - Google search using search terms - Documents collected directly from experts, decision-makers, and other immunization stakeholders |
| --- | --- |

We will catalog all identified documents in our Excel spreadsheet and apply the same inclusion or exclusion criteria (Table 3) to assess relevance based on a review of the titles. Documents assessed to be relevant will be included.

**Step 4: Quality assessment**

The goal of the quality assessment is to ensure the evidentiary claims are reliable and contribute meaningfully to answering our research questions. We propose using a checklist to concurrently appraise the quality of quantitative, qualitative, and mixed-methods primary studies included in the review, drawing from the Mixed Methods Appraisal Tool [10]. The quality scores will be presented in the gap map described in Step 6 so that readers and decision-makers can view the quality of the included evidence at a glance.

**Step 5: Data extraction**

We will import the included documents into Atlas.Ti, a qualitative coding software. We will create a qualitative coding tree that includes each of the final contextual factors, mechanisms, and outcomes (C-M-O) identified in the TOC, as well as the hypothesized causal relationships between them. Reviewers will read each document and apply the codes to relevant text segments. Through this process, we will organize all the evidence into the TOC C-M-O dimensions.

**Step 6: Data synthesis and evidence gap map**

The aim of Step 6 is to transform the coded text segments from Step 5 into meaningful conclusions. We will organize the results in an evidence gap map to help visualize the *quantity* and *strength* of evidence for each mechanism-to-outcome relationship in a matrix format. We will:

1. Define the gap map rows (intervention mechanisms) and columns (intermediate and final outcomes) based on our final TOC.
2. Export the text for each code from Atlas.Ti (from Step 5) and carefully read the text to gain a deeper understanding of how the evidence supports or refutes that particular TOC mechanism, context, outcome, or relationships between mechanism/context/outcome.
3. Develop a narrative summary of the evidence for each mechanism-outcome relationship (e.g., each cell in the gap map with available evidence), noting how context may alter the observed or expected causal pathways. At this step, we will also list the number of documents contributing to this statement, as well as the quality appraisal score of the strength of the included evidence.

**References**

1. Pawson R, Greenhalgh T, Harvey G, Walshe K. Realist review-a new method of systematic review designed for complex policy interventions. J Health Serv Res Policy. 2005;10(1_suppl):21–34.

2. Robert E, Ridde V, Marchal B, Fournier P. Protocol: a realist review of user fee exemption policies for health services in Africa. BMJ Open. 2012;2(1):e000706.

3. Gough D, Thomas J, Oliver S. Clarifying differences between review designs and methods. Syst Rev. 2012;1(1):28.

4. Nutley T, Reynolds H. Improving the use of health data for health system strengthening. Glob Health Action. 2013;6(1):2001.

5. Aqil A, Lippeveld T, Hozumi D. PRISM framework: a paradigm shift for designing, strengthening and evaluating routine health information systems. Health Policy Plan. 2009;24(3):217–228.

6. Langer L, Tripney J, Gough D. The Science of Using Science: Researching the Use of Research Evidence in Decision-Making. London: EPPI-Centre, Social Science Research Unit, UCL Institute of Education, University College London.; 2016.

7. Zuske, Meike, Jarrett, Caitlin, Auer, Christian, Bosch-Capblanch, Xavier, Oliver, Sandy. Health Information System Use: framework synthesis (draft). Swiss TPH; 2017.

8. World Health Organization. Framework for Partner Collaboration to Strengthen Immunization and Surveillance Data for Decision-making (draft). World Health Organization; 2017.

9. PATH. Defining and Building a Data Use Culture (draft). PATH; 2017.

10. Pace R, Pluye P, Bartlett G, Macaulay AC, Salsberg J, Jagosh J, et al. Testing the reliability and efficiency of the pilot Mixed Methods Appraisal Tool (MMAT) for systematic mixed studies review. Int J Nurs Stud. 2012;49(1):47–53.

11. Michie S, van Stralen MM, West R. The behaviour change wheel: A new method for characterizing and designing behaviour change interventions. Implementation Sci. 2011;6:42.
