# Supplementary material for: A realist systematic review of evidence from low- and middle-income countries of interventions to improve immunization data use
Source: BMC Health Serv Res. 2021 Jul 8;21:672. doi: 10.1186/s12913-021-06633-8 (PMC8268169; doi:10.1186/s12913-021-06633-8)
Supplement: Supplementary file 2 — Additional file 2: [file 12913_2021_6633_MOESM2_ESM.docx]

Appendix B. List of included articles

Each included article was assigned to a primary intervention category. Reviewers appraised the quality of immunization data use articles categorized as *evidence* using the Mixed Methods Appraisal Tool (MMAT) checklist, which translates into a percentage score. ‘Strong’-quality studies scored 75-100%; ‘Moderate’-quality studies scored 50-74%; ‘Weak’-quality studies scored 0-49%.^19^

| **Author(s)** | **Year** | **Intervention category** | **Health sector** | **Evidence from LMIC or HIC** | **Grey or peer-reviewed literature** | **Evidence or  promising strategy** | **MMAT Score** |
| --- | --- | --- | --- | --- | --- | --- | --- |
| Aman et al.^39^ | 2016 | Logistics management information systems | Immunization | LMIC | Grey | Evidence | 86% |
| Aqil^70^ | 2008 | Data quality assessments | Other | LMIC | Grey | Promising strategy | N/A |
| Beltrami et al.^91^ | 2017 | Supportive supervision, mentorship, and on-the-job learning | Other | HIC | Peer-reviewed | Evidence | N/A |
| Bosch-Capblanch and Garner^89^ | 2008 | Supportive supervision, mentorship, and on-the-job learning | Other | LMIC | Peer-reviewed | Evidence | N/A |
| Bosch-Capblanch et al.^63^ | 2009 | Data quality assessments | Immunization | LMIC | Peer-reviewed | Evidence | 100% |
| Braa et al.^72^ | 2012 | Data review meetings | Other | LMIC | Peer-reviewed | Evidence | N/A |
| Campbell et al.^103^ | 2014 | mHealth | Other | LMIC | Peer-reviewed | Evidence | N/A |
| CDC^93^ | 2018 | Supportive supervision, mentorship, and on-the-job learning | Immunization | LMIC | Grey | Evidence | 57% |
| CDC^99^ | Unknown | Training | Other | LMIC | Grey | Evidence | N/A |
| CDC^29^ | 2016 | Electronic immunization registries | Immunization | LMIC | Grey | Evidence | 60% |
| CDC^54^ | 2016 | Decision support systems | Immunization | LMIC | Grey | Promising strategy | N/A |
| CDC^92^ | 2018 | Supportive supervision, mentorship, and on-the-job learning | Immunization | LMIC | Grey | Promising strategy | N/A |
| CDC^98^ | 2018 | Training | Other | LMIC | Grey | Evidence | N/A |
| Chandani et al.^77^ | 2016 | Peer learning networks | Other | LMIC | Peer-reviewed | Evidence | N/A |
| Chandani et al.^111^ | 2014 | Other / multicomponent interventions | Other | LMIC | Peer-reviewed | Evidence | N/A |
| Chowdhury^30^ | 2018 | Electronic immunization registries | Immunization | LMIC | Grey | Evidence | 67% |
| Chowdhury^31^ | 2018 | Electronic immunization registries | Immunization | LMIC | Grey | Evidence | 50% |
| Cibulskis et al.^47^ | 1995 | Decision support systems | Immunization | LMIC | Peer-reviewed | Evidence | 89% |
| Courtenay-Quirk et al.^94^ | 2016 | Training | Other | LMIC | Peer-reviewed | Evidence | N/A |
| Dehnavieh et al.^42^ | 2018 | Health management information systems | Other | LMIC | Peer-reviewed | Evidence | N/A |
| Dell et al.^23^ | 2012 | Electronic immunization registries | Immunization | LMIC | Peer-reviewed | Evidence | 43% |
| Escalante et al.^106^ | 2016 | mHealth | Immunization | LMIC | Grey | Promising strategy | N/A |
| Garrib et al.^43^ | 2008 | Health management information systems | Other | LMIC | Peer-reviewed | Evidence | N/A |
| Gilbert et al.^34^ | 2017 | Logistics management information systems | Immunization | LMIC | Peer-reviewed | Evidence | 80% |
| Gimbel et al.^64^ | 2017 | Data quality assessments | Other | LMIC | Peer-reviewed | Evidence | N/A |
| GlaxoSmithKline^105^ | 2016 | mHealth | Immunization | LMIC | Grey | Promising strategy | N/A |
| Groom et al.^21^ | 2015 | Electronic immunization registries | Immunization | HIC | Peer-reviewed | Evidence | 100% |
| Haidar et al.^36^ | 2017 | Logistics management information systems | Immunization | LMIC | Grey | Evidence | 54% |
| He et al.^87^ | 2014 | Supportive supervision, mentorship, and on-the-job learning | Immunization | LMIC | Peer-reviewed | Evidence | 80% |
| Heidebrecht et al.^109^ | 2014 | Other / multicomponent interventions | Immunization | HIC | Peer-reviewed | Promising strategy | N/A |
| Jain et al.^49^ | 2015 | Decision support systems | Immunization | LMIC | Peer-reviewed | Evidence | 71% |
| Jandee^100^ | 2016 | mHealth | Immunization | LMIC | Peer-reviewed | Promising strategy | N/A |
| JSI^83^ | 2016 | Peer learning networks | Other | LMIC | Grey | Evidence | N/A |
| JSI^84^ | Unknown | Peer learning networks | Immunization | LMIC | Grey | Promising strategy | N/A |
| JSI^117^ | 2016 | Other / multicomponent interventions | Immunization | LMIC | Grey | Promising strategy | N/A |
| JSI^79^ | 2016 | Peer learning networks | Immunization | LMIC | Grey | Promising strategy | N/A |
| JSI^112^ | 2017 | Other / multicomponent interventions | Other | LMIC | Grey | Promising strategy | N/A |
| JSI^57^ | 2017 | Decision support systems | Immunization | LMIC | Grey | Evidence | 43% |
| JSI^81^ | 2017 | Peer learning networks | Other | LMIC | Grey | Promising strategy | N/A |
| Karuri et al.^1^ | 2014 | Health management information systems | Other | LMIC | Peer-reviewed | Evidence | N/A |
| Keny et al.^22^ | 2013 | Electronic immunization registries | Immunization | LMIC | Peer-reviewed | Evidence | 100% |
| Kihuba et al.^44^ | 2014 | Health management information systems | Other | LMIC | Peer-reviewed | Evidence | N/A |
| Kindoli^25^ | 2017 | Electronic immunization registries | Immunization | LMIC | Grey | Evidence | 85% |
| LaFond et al.^74^ | 2012 | Data review meetings | Immunization | LMIC | Grey | Evidence | 86% |
| Ledikwe et al.^97^ | 2013 | Training | Other | LMIC | Peer-reviewed | Evidence | N/A |
| Li^78^ | 2017 | Peer learning networks | Other | LMIC | Grey | Evidence | N/A |
| Macdonald^26^ | 2018 | Electronic immunization registries | Immunization | LMIC | Grey | Evidence | 86% |
| Moja et al.^50^ | 2014 | Decision support systems | Other | HIC | Peer-reviewed | Evidence | N/A |
| MSHP du République de Côte d'Ivoire^66^ | 2017 | Data quality assessments | Immunization | LMIC | Grey | Evidence | 83% |
| Mutemwa^46^ | 2006 | Health management information systems | Other | LMIC | Peer-reviewed | Evidence | N/A |
| Muthee et al.^61^ | 2018 | Data quality assessments | Other | LMIC | Peer-reviewed | Evidence | N/A |
| Negandhi et al.^102^ | 2016 | mHealth | Immunization | LMIC | Peer-reviewed | Evidence | 67% |
| Nguyen et al.^20^ | 2017 | Electronic immunization registries | Immunization | LMIC | Peer-reviewed | Evidence | 79% |
| Nshunju^35^ | 2018 | Logistics management information systems | Immunization | LMIC | Grey | Evidence | 75% |
| Nutley et al.^2^ | 2014 | Other / multicomponent interventions | Other | LMIC | Peer-reviewed | Evidence | N/A |
| Nutley et al.^53^ | 2013 | Decision support systems | Other | LMIC | Peer-reviewed | Evidence | 71% |
| O'Hagan et al.^62^ | 2017 | Data quality assessments | Other | LMIC | Peer-reviewed | Evidence | N/A |
| PAHO^67^ | 2009 | Data quality assessments | Immunization | LMIC | Grey | Promising strategy | N/A |
| PAHO^68^ | 2011 | Data quality assessments | Immunization | LMIC | Grey | Promising strategy | N/A |
| PAHO^56^ | 2016 | Decision support systems | Immunization | HIC | Grey | Promising strategy | N/A |
| PAHO^69^ | 2018 | Data quality assessments | Immunization | LMIC | Grey | Promising strategy | N/A |
| PAHO^71^ | 2014 | Data quality assessments | Immunization | HIC | Grey | Promising strategy | N/A |
| PAHO^80^ | 2012 | Peer learning networks | Immunization | LMIC | Grey | Promising strategy | N/A |
| Pappaioanou et al.^95^ | 2003 | Training | Immunization | LMIC | Peer-reviewed | Evidence | 43% |
| PATH^85^ | 2015 | Peer learning networks | Immunization | LMIC | Grey | Promising strategy | N/A |
| PATH^82^ | 2018 | Peer learning networks | Immunization | LMIC | Grey | Promising strategy | N/A |
| Poy et al.^52^ | 2017 | Decision support systems | Immunization | LMIC | Peer-reviewed | Promising strategy | N/A |
| Ramanujapuram and Akkihal^101^ | 2014 | mHealth | Immunization | LMIC | Peer-reviewed | Evidence | 100% |
| Robinson et al.^75^ | 2001 | Peer learning networks | Immunization | LMIC | Peer-reviewed | Evidence | N/A |
| Rolle et al.^96^ | 2011 | Training | Other | LMIC | Peer-reviewed | Promising strategy | N/A |
| Rowe et al.^88^ | 2005 | Supportive supervision, mentorship, and on-the-job learning | Other | LMIC | Peer-reviewed | Evidence | N/A |
| Rowe et al.^108^ | 2018 | Other / multicomponent interventions | Other | LMIC | Peer-reviewed | Evidence | N/A |
| Rowe^116^ | 2018 | Other / multicomponent interventions | Other | LMIC | Grey | Evidence | N/A |
| Scott et al.^65^ | 2017 | Data quality assessments | Immunization | LMIC | Peer-reviewed | Promising strategy | N/A |
| Shieshia et al.^76^ | 2014 | Peer learning networks | Other | LMIC | Peer-reviewed | Evidence | N/A |
| Shifo Foundation^115^ | 2018 | Other / multicomponent interventions | Immunization | LMIC | Grey | Promising strategy | N/A |
| Shifo Foundation^118^ | 2018 | Other / multicomponent interventions | Immunization | LMIC | Grey | Promising strategy | N/A |
| Shimp et al.^73^ | 2017 | Data review meetings | Immunization | LMIC | Peer-reviewed | Evidence | 83% |
| Sowe^32^ | 2018 | Electronic immunization registries | Immunization | LMIC | Grey | Evidence | 67% |
| Srinivasan^104^ | 2017 | mHealth | Immunization | LMIC | Grey | Promising strategy | N/A |
| Sudhof et al.^110^ | 2013 | Other / multicomponent interventions | Other | LMIC | Peer-reviewed | Promising strategy | N/A |
| Trumbo et al.^24^ | 2018 | Electronic immunization registries | Immunization | LMIC | Peer-reviewed | Evidence | 83% |
| UI-FHS^114^ | 2015 | Other / multicomponent interventions | Immunization | LMIC | Grey | Promising strategy | N/A |
| Vasan et al.^90^ | 2017 | Supportive supervision, mentorship, and on-the-job learning | Other | LMIC | Peer-reviewed | Evidence | N/A |
| Vedanthan et al.^51^ | 2015 | Decision support systems | Other | LMIC | Peer-reviewed | Evidence | N/A |
| Villag Reach^58^ | 2017 | Decision support systems | Immunization | LMIC | Grey | Promising strategy | N/A |
| Village Reach^38^ | 2015 | Logistics management information systems | Immunization | LMIC | Grey | Promising strategy | N/A |
| Wagenaar et al.^60^ | 2015 | Data quality assessments | Immunization | LMIC | Peer-reviewed | Evidence | 100% |
| Ward et al.^86^ | 2017 | Supportive supervision, mentorship, and on-the-job learning | Immunization | LMIC | Peer-reviewed | Evidence | 67% |
| Weeks et al.^48^ | 2000 | Decision support systems | Immunization | LMIC | Peer-reviewed | Evidence | 33% |
| Westley et al.^33^ | 2014 | Electronic immunization registries | Immunization | HIC | Grey | Promising strategy | N/A |
| Whelan^119^ | 2018 | Other / multicomponent interventions | Immunization | LMIC | Grey | Promising strategy | N/A |
| Wheldon^113^ | 2015 | Other / multicomponent interventions | Immunization | LMIC | Grey | Promising strategy | N/A |
| WHO^107^ | 2013 | mHealth | Immunization | LMIC | Grey | Promising strategy | N/A |
| WHO^28^ | 2013 | Electronic immunization registries | Immunization | LMIC | Grey | Evidence | 33% |
| WHO^55^ | 2007 | Decision support systems | Immunization | LMIC | Grey | Evidence | 57% |
| WHO^37^ | 2013 | Logistics management information systems | Immunization | LMIC | Grey | Evidence | 71% |
| WHO^40^ | 2013 | Logistics management information systems | Immunization | LMIC | Grey | Evidence | 71% |
| WHO^41^ | 2013 | Logistics management information systems | Immunization | LMIC | Grey | Evidence | 71% |
| WHO^59^ | 2018 | Decision support systems | Other | LMIC | Grey | Evidence | N/A |
| Wickremasinghe et al.^45^ | 2016 | Health management information systems | Other | LMIC | Peer-reviewed | Evidence | N/A |
| Zulu^27^ | 2018 | Electronic immunization registries | Immunization | LMIC | Grey | Evidence | 85% |

Abbreviations: HIC, high-income country; LMIC, low- and middle-income country; MMAT, Mixed Methods Appraisal Tool; N/A, not applicable.
