# Supplementary material for: A realist systematic review of evidence from low- and middle-income countries of interventions to improve immunization data use
Source: BMC Health Serv Res. 2021 Jul 8;21:672. doi: 10.1186/s12913-021-06633-8 (PMC8268169; doi:10.1186/s12913-021-06633-8)
Supplement: Supplementary file 3 — Additional file 3: [file 12913_2021_6633_MOESM3_ESM.docx]

Appendix C. Evidence synthesis tables

**Table 1 Electronic immunization registries**

| **Hypothesized mechanisms** | **Contextual factors that affected how the intervention worked and intervention components hypothesized to support data use** | |
| --- | --- | --- |
| **Access and availability**  Data are easier to extract and more accessible to the user  **Data quality**  Enhanced by built­ in data validation features and the ability to track children across multiple facilities  **Opportunity**  **Workflow** processes are simplified and streamlined, and the need for numerous paper records is eliminated | **Capability factors**   - Computer literacy of health workers - Knowledge of how to use data for action   **Motivation factors**   - Heath workers' perception of improved data quality - The extent to which health workers must perform parallel data entry - Simplification and streamlining of workflow and reporting processes - Availability of support from mentors and higher-level management   **Opportunity factors**   - Degree to which the burden of data entry from paper records is minimized - Extent to which higher- level managers' expectations around data quality and use are clear - Adequacy of human resources to manage associated administrative burden - Stability of electricity and Internet connectivity - Interoperability with the broader HIS and vaccine stock management system - Extent to which country has a registry culture in which health workers already register children in a paper­ book or card system   **Intervention functionalities/components**   - Automatic generation of monthly immunization reports and lists of children due for vaccination - Automatic text message vaccination reminders to caretakers - Longitudinal tracking of vaccination history for individual children - Data storage and aggregation - Routine health indicator data collection and management - Application of technological solutions to facilitate the digitization of paper records - Leveraging of complementary activities to reinforce analytic capacity and data use | |
|  |  |  |
|  |  |  |
|  |  |  |
|  |  |  |
|  |  |  |
|  |  |  |
|  |  |  |
|  |  |  |
|  |  |  |
|  |  |  |
| **Data use outcome** | **Evidence of the intervention's effect on data use** | **Strength of the evidence** |
| **Intermediate outcomes** | **We are uncertain about the effect on data availability**   - Four studies and one systematic review found that data were more available.^20,21,22,25^ - In three studies, data availability was undermined by inconsistent use of the EI R, which resulted from challenges with operationalizing electronic data entry.^26,28,29^ | Very low |
|  | **Increases data quality**   - Five studies found improvements in data quality, including reduced barriers to data use related to data quality, more accurate data entry, and a perception among health workers of higher-quality data in the EIR.^20,22,25,26,27^ - One case study found challenges with the quality of denominator data, which led to overestimating coverage.^24^ | Moderate |
|  | **Increases data synthesis, review, analysis, and interpretation**   - Two studies found self-reported increases in data synthesis and review by health workers and increases in their ability to analyze and interpret data, such as identifying defaulters, areas of low coverage, and vaccine stock levels.^25,27^ - Three studies found that nurses were confident with synthesizing data using the EIR.^25,26,27^ | Moderate |
|  | **mHealth tools used to digitize paper immunization records contribute to improved data quality**   - One case study found that digitized child immunization history records were more complete than manually entered records.^100^ - Three evaluations and one study found that scanning technology was able to accurately digitize data from paper forms and reduce the amount of time spent on manual data entry.^23,30,31,32^ - One mixed-methods study found no difference in data quality, and improvements in the timeliness of data entry were mixed.^26,109^ | Low |
| **Data use by health facilities** | **We are uncertain about the effect on data use by health facilities**   - Two studies found a self-reported increase in taking action in response to their data^25,27,^ and one study found no significant change between baseline and midline, although it may have been too early to detect significant changes in data use behavior.^26^ - In one study, facility health workers could express a plan for data use, but others could not identify ways to use data for action.^29^ | Very low |
| **Data use by health districts** | **Improves data use and emphasis on data by health districts**   - In one study, district staff reported using EIR data in data review meetings to make decisions.^27^ - In the same study and one other study, facility health workers reported an improved emphasis on data quality and use by higher-level health staff, but clarity regarding their roles around data quality and use could have been improved.^25,27^ | Low |
| **Data use by national program** | **Uncertain**   - No studies were identified that reported this outcome. | No  evidence |

**Table 2 Logistics management information systems**

| **Hypothesized mechanisms** | **Contextual factors that affected how the intervention worked and intervention components hypothesized to support data use** | | |
| --- | --- | --- | --- |
| **Access and availability**  Data are available in real time to users at multiple levels for more timely action  **Data quality**  Streamlined data entry and secure data storage  **Structure and process**  Harness data management technology to systematize decision-making processes  **Opportunity**  **Workflow** Processes are simplified and streamlined | **Capability factors**   - Easy-to-understand visualizations   **Motivation factors**   - Degree to which complementary platforms (e.g., data review meetings) are leveraged to support reviewing and interpreting data and problem-solving - Communication between key supply chain collaborators (e.g., logisticians, EPI manager, facility staff, etc.) - Extent to which design responds to data user requirements and expectations - Timely, accurate, and accessible data   **Opportunity factors**   - Degree to which the burden of data entry from paper records is minimized - Extent to which human resource needs are met (e.g., dedicated logisticians) - Interoperability with the broader HIS - Stability of electricity and Internet connectivity - Tool's ability to work seamlessly across web and mobile software devices   **Intervention functionalities/components**   - Supply chain data available to decision-makers in real time - Built-in data dashboard visualization and analytics - Vaccine shipment tracking (successful operationalization was contingent on buy-in from national-level users) - Vaccine stock management - Automated monthly reporting on administered vaccines | | |
|  |  |  |  |
|  |  |  |  |
|  |  |  |  |
|  |  |  |  |
|  |  |  |  |
|  |  |  |  |
|  |  |  |  |
|  |  |  |  |
|  |  |  |  |
| **Data use outcome** | **Evidence of the intervention's effect on data use** | | **Strength of the evidence** |
| **Intermediate outcomes** | **Increases data quality and availability**   - Five studies found substantial improvements in the availability and quality of vaccine stock records at both regional and district levels.^34,36,37,39,41^ - One quasi-experimental implementation research study found higher data consistency in the intervention districts compared with nonintervention (paper based) districts after one year of implementation; however, these differences were not statistically significant (p = .20).^35^ | Moderate | |
|  | **Increases data synthesis, review, analysis, and interpretation**   - One study found self-reported data, confirmed by observational data, of improved skills and knowledge related to the analysis and interpretation of monthly supply chain data by provincial and district managers.^39^ | Low | |
|  | **mHealth solutions applied to LMIS interventions contribute to increases in data availability and accessibility**   - Three studies found improvements in EVM indicators and supply chain performance due to greater availability of high-quality, real-time data for decision-making.^76,101,102^ | Low | |
| **Data use by health facilities** | **Uncertain**   - No studies were identified that reported this outcome. | No  evidence | |
| **Data use by health districts** | **Improves data use for vaccine stock management by health districts**   - One mixed-methods study found evidence of increased use of data for supply chain management, including improvements in EVM indicators related to data use in vaccine forecasting and wastage reporting: provincial and district managers self-reported that vLMIS improved their use of data to make decisions on vaccine stocking and decisions related to monitoring and supervising facilities.^39^ - One quasi-experimental study found a quicker response to stockouts and cold chain equipment breakdown reports between baseline and end line (responses within 24 hours increased from 20% to 87% and from 10% to 59%, respectively).^36^ - Program data from one intervention showed an improvement in vaccine delivery intervals and reports of data influencing action taken to resolve vaccine delivery delays.^38^ | Moderate | |
| **Data use by national program** | **Uncertain**   - No studies were identified that reported this outcome. | No  evidence | |

**Table 3 Health management information systems**

| **Hypothesized mechanisms** | **Contextual factors that affected how the intervention worked and intervention components hypothesized to support data use** | | |
| --- | --- | --- | --- |
| **Access and availability**  data are available in real time to users at multiple levels for more timely action  **Data quality**  automatic data validation features and secure data storage | **Capability factors**   - Extent to which users are supported through training, on-site mentorship, and supportive supervision - Decision-makers' data analysis capacity - Utilization of tools/frameworks for structured decision-making   **Motivation factors**   - Health workers' attitudes and interest in reporting in and using new systems - Decision-maker autonomy - Extent to which clinic staff receive feedback on the data they submit   **Opportunity factors**   - Availability of appropriately skilled personnel - Quality and availability of data in the system   **Intervention functionalities/components**   - Computerized record keeping and data aggregation - Data accessible to decision­ makers in real time - Embedded data validation checks - Automated report generation | | |
|  |  |  |  |
|  |  |  |  |
|  |  |  |  |
|  |  |  |  |
|  |  |  |  |
|  |  |  |  |
|  |  |  |  |
|  |  |  |  |
|  |  |  |  |
| **Data use outcome** | **Evidence of the intervention's effect on data use** | | **Strength of the evidence** |
| **Intermediate outcomes** | **Contributes to improved data quality and availability**   - One systematic review and a review of seven cases studies found improvements in data quality and completeness; greater visibility of facility performance appeared to incentivize improvements.^1,42^ | Moderate | |
|  | **Does not contribute to improvements in data analysis, interpretation, and review as a stand-alone intervention**   - One nonexperimental, mixed-methods study found no evidence of improved data analysis, interpretation, and/or review at the facility level, owing to the absence of feedback and support mechanisms.^43^ | Low | |
|  | **Greater data use improves data quality**   - One systematic review and a review of seven case studies found that greater data use led to greater ownership of and demand for high-quality data.^1,42^ | Moderate | |
| **Data use by health facilities** | **Does not lead to improved data use**   - Two nonexperimental, mixed-methods studies found low utilization of HMIS data at the service-delivery level when feedback and other support mechanisms were absent from higher levels.^43,44^ | Low | |
| **Data use by health districts** | **Improves data use by health districts**   - One systematic review found that health districts were using data for facility monitoring and performance improvement and for district implementation planning and prioritization.^45^ - One multi-country case study found evidence of data use in four out of seven countries examined.^1^ - One qualitative case study found evidence of HMIS data use for decision-making, in addition to verbal, observational, and experiential sources of information.^46^ | Moderate | |
| **Data use by national program** | **Uncertain**   - No studies were identified that reported this outcome. | No evidence | |

**Table 4 Decision support systems (e.g., CDSS, monitoring charts, dashboards, and HBRs)**

| **Hypothesized mechanisms** | **Contextual factors that affected how the intervention worked and intervention components hypothesized to support data use** | |
| --- | --- | --- |
| **Structure and process**  Strengthen decision-making structures and processes  **Skills**  Support data analysis, helping users to transform data into actionable information | **Capability factors**   - Utilization of user specific DHIS2 training modules - Mobilization of human resource support to provide hands-on learning and mentoring   **Motivation factors**   - Degree to which data analysis and use are reinforced by consistent feedback through both training and supportive supervision - Integration with existing systems and workflows (e.g., leveraged by data review meetings)   **Opportunity factors**   - Completeness and accuracy of the underlying data   **Intervention functionalities/components**   - Data aggregation from multiple sources - Data synthesis and visualization - Automated analysis of data for easy interpretation - Automated report generation - Tailored analysis in response to specific programmatic questions | |
|  |  |  |
|  |  |  |
|  |  |  |
|  |  |  |
|  |  |  |
|  |  |  |
|  |  |  |
|  |  |  |
| **Data use outcome** | **Evidence of the intervention's effect on data use** | **Strength of the evidence** |
| **Intermediate outcomes** | **Improves data quality, analysis, synthesis, interpretation, and review**   - Two evaluations with nonexperimental study designs, one case study, and one project report found that simple, paper-based immunization monitoring charts and dashboards increased awareness and tracking of immunization coverage and led to improvements in data quality.^48,49,54,55^ - One mixed-methods evaluation found that CDSSs were more likely to improve analysis and interpretation of data in low-performing regions.^47^ | Moderate |
| **Data use by health facilities** | **Improves data use by communities and health facilities**   - One evaluation with a nonexperimental study design and one project report found that facilities used monitoring charts to review whether they were meeting targets, respond to high dropout rates and low vaccine coverage, and follow up on defaulters.^49,54^ | Moderate |
| **Data use by health districts** | **Monitoring charts and dashboards improve data use by health districts to bolster facility performance and data quality**   - One project report found that data were used by health districts for facility performance tracking to prioritize facilities for supportive super vision. The same report also found that data use led to improvements in data quality.^54^   *In the evidence from the other health sectors:*   - One qualitative evaluation found evidence that a Microsoft Excel-based data dashboard, tailored to answer specific programmatic questions, was used by district health managers to monitor and address facility performance problems and to improve data quality.^42^ | Moderate |
|  | **We are uncertain about the effect of CDSSs on data use**   - One mixed-methods evaluation found that district health officers in low­ performing regions were more likely to use CDSS to give feedback to health facilities.^47^ - One systematic review from 28 RCTs in high-income countries found little to no difference in clinical outcomes.^50^ - One feasibility study of a tablet based CDSS for clinical care of patients with hypertension reported perceptions among nurses that the tool made patient encounters easier and improved the quality of care.^51^ | Very low |
| **Data use by national program** | **Uncertain**   - No studies were identified that reported this outcome. | No evidence |

**Table 5 Data quality assessments**

| **Hypothesized mechanisms** | **Contextual factors that affected how the intervention worked and intervention components hypothesized to support data use** | |
| --- | --- | --- |
| **Data quality**  Complete and accurate data more likely to be used for sound decision-making | **Capability factors**   - Extent to which staff have the skills and training needed to assess data quality properly   **Motivation factors**   - Extent to which the intervention is paired with feedback and skills reinforcement through targeted training, supervision, and feedback meetings   **Opportunity factors**   - Ratio of human resources for health to patients at the facility level   **Intervention functionalities/components**   - Standardized methodology for systematically assessing and quantifying data quality - Support for self-assessment of data quality - Expanded scope of DQS Plus methodologies for a more holistic assessment of IIS performance - Production of actionable DQIPs facilitated by methodology | |
|  |  |  |
|  |  |  |
|  |  |  |
|  |  |  |
|  |  |  |
|  |  |  |
|  |  |  |
|  |  |  |
| **Data use outcome** | **Evidence of the intervention's effect on data use** | **Strength of the evidence** |
| **Intermediate outcomes** | **Leads to improvements in data quality**   - One time-series observational study found statistically significant improvements in data concordance, and three reports on repeat DQS found an increase in the number of facilities with a satisfactory verification factor.^60,66,67,68^ - One review of data quality in 41 countries found that data quality improved (verification factor and quality score) in 6 countries that conducted repeat DQAs.^63^   *In the evidence from other health sectors:*   - One nonexperimental study found improved data quality, including a decline in missing data (from 31% to13%) and an increase in data concordance (from 59% to 68%) at the facility level between baseline and follow-up routine data quality assessments.^61^ - One experimental study found that data use by health facilities was associated with improved data avail ability (p = .04) and data completeness (p = .02) but not with higher data accuracy.^62^ | Moderate to high |
| **Data use by health facilities** | **Improves health facilities' use of data to improve data quality**   - Six studies, including five with a nonexperimental design and one with an experimental design that demonstrated improvements in data quality, suggest that DQAs prompted health facilities to use data to improve data quality.^60,61,62,66,67,68^ | Moderate |
|  | **Improvements in data quality lead to greater data use by health facilities**   - One time-series observational study found that facilities with high-quality data were less likely to have stockouts.^60^ | Low |
| **Data use by health districts** | **Uncertain**   - No studies were identified that reported this outcome. | No  evidence |
| **Data use by national program** | **Encourages data use by the national program to inform vaccine strategies and policies**   - One study reported anecdotes that the DQIP led to concrete actions taken by the national program in two countries to improve data quality through changes in vaccination program strategies and policies.^65^ | Very low |

**Table 6 Data review meetings**

| **Hypothesized mechanisms** | **Contextual factors that affected how the intervention worked and intervention components hypothesized to support data use** | |
| --- | --- | --- |
| **Demand**  Foster a culture of data use by building knowledge­ seeking and data­sharing behaviors  **Skills**  Leverage peer learning and knowledge sharing to build skills and confidence in data analysis  **Structure and process**  Support and reinforce country processes that build data use into the decision­ making process  **Motivation**  Demonstrate how data can be used to improve program performance | **Capability factors**   - Extent to which the intervention is paired with activities that further support data analysis and provide follow-up or feedback loops - Leveraging quality improvement methodologies for a structured approach to data analysis and problems-solving - Extent to which review meetings build progressively upon previous meeting recommendations and discussions to reinforce and supplement learning and practices   **Motivation factors**   - Focus on team-oriented problem-solving and learning - Extent to which data review examines data completeness. data verification, and interpretation of performance data - Equal representation from data users and data producers   **Opportunity factors**   - Likelihood of adoption and sustainability given the intervention's fit within existing immunization program processes and budget   **Intervention functionalities/components**   - Convening immunization stakeholders at multiple levels - Communication and performance feedback for health care providers - Peer exchange and problem-solving - Application of quality improvement methodologies | |
|  |  |  |
|  |  |  |
|  |  |  |
|  |  |  |
|  |  |  |
|  |  |  |
|  |  |  |
|  |  |  |
| **Data use outcome** | **Evidence of the intervention's effect on data use** | **Strength of the evidence** |
| **Intermediate outcomes** | **Improves data quality when combined with supportive activities in the context of broader efforts to improve health information infrastructure**   - One longitudinal case study reported a reduction in the proportion of health facilities with disparities among vaccine coverage indicators.^73^ - One case study reported significant improvements in data quality due in part to quarterly data review meetings implemented in the context of broader efforts to strengthen quality and use of HMIS and DHIS2 data in Tanzania.^72^ | Low |
|  | **Improves data interpretation and review**   - One longitudinal case study reported that after multiple rounds of review meetings, health workers were better able to interpret immunization data and correctly complete monitoring charts.^73^ | Low |
| **Data use by health facilities** | **Uncertain**   - No studies were identified that reported this outcome. | No evidence |
| **Data use by health districts** | **Improves data use by health districts**   - One longitudinal case study reported an anecdotal example of a health district using data to resolve a problem identified during the QRM.^73^ - One case study reported a number of instances of improved data use (and quality) such as follow-up on immunization defaulters, better understanding of denominator issues, and increased tracing of indicators and targets.^72^ | Low |
| **Data use by national program** | **Uncertain**   - No studies were identified that reported this outcome | No evidence |

**Table 7 Peer learning networks**

| **Hypothesized mechanisms** | **Contextual factors that affected how the intervention worked and intervention components hypothesized to support data use** | |
| --- | --- | --- |
| **Demand**  Cultivate a culture of data use and empower health workers  **Skills**  Reinforced through information and knowledge exchange  **Motivation**  Offer support and peer examples of data use successes | **Capability factors**   - Leveraging quality improvement methodologies for a structured approach to data analysis and problem-solving - Leveraging tools that facilitate data management, analysis, and visualization (e.g., data dashboards)   **Motivation factors**   - Providing opportunities for working one-to-one, in a non-threatening atmosphere, with more experienced peers - Willingness to share data based on concerns that poor quality data would reflect negatively on individual performance   **Opportunity factors**   - Multidisciplinary nature of the network or team (e.g., involving immunization stakeholders across departments, functions, and levels)   **Intervention functionalities/components**   - Information and knowledge exchange - Collective problem-solving using structured approaches - Increasing collaboration, communication, and coordination among immunization stakeholders at multiple levels and functions | |
|  |  |  |
|  |  |  |
|  |  |  |
|  |  |  |
|  |  |  |
|  |  |  |
|  |  |  |
| **Data use outcome** | **Evidence of the intervention's effect on data use** | **Strength of the evidence** |
| **Intermediate outcomes** | **Improves data review, analysis, and interpretation**   - One mixed-methods study found a self-reported increase in health worker knowledge, motivation, and skills related to data use. Two other projects reported M&E results showing anecdotal evidence of health workers at facility and district levels working collaboratively to review, analyze, and interpret data.^25,26,27^ - Two observational studies found that QITs met regularly to review stock data, identify challenges, and determine solutions (such as moving stock between over- and under-stocked facilities).^83,84^ | Low |
| **Data use by health facilities** | **Improves use of data to monitor vaccine supply and cold chain**   - One observational study found that facilities that received the intervention had fewer stockouts.^83^ | Low |
| **Data use by health districts** | **Uncertain**   - No studies were identified that reported this outcome. | No  evidence |
| **Data use by national program** | **Improves data use in decision-making by the national program**   - In one survey, national-level network participants reported becoming more data oriented in their work and making decisions based on data.^82^ | Low |

**Table 8 Supportive supervision, mentorship, and on-the-job training**

| **Hypothesized mechanisms** | **Contextual factors that affected how the intervention worked and intervention components hypothesized to support data use** | | |
| --- | --- | --- | --- |
| **Skills**  Build data analysis skills and knowledge  **Capability**  Build ability to transform data into actionable information | **Capability factors**   - Extent to which supervision and mentorship are site specific (i.e., tailored to the specific gaps in skills and data management, analysis, and use practices identified in assessments) - Application of audit-and­ feedback techniques   **Motivation factors**   - Extent to which supervision is individualized and open - Two-way flow of information between supervisor and community health worker - Extent to which expectations for data use are clear and feedback is consistent - Frequency of routine follow-up (including both oral and written feedback) - Extent to which health workers are empowered to engage in proactive problem-solving   **Opportunity factors**   - Management and leadership clarity on roles and expectations related to data analysis and use - Degree to which individuals across multiple levels of the health system are connected and engaged - Integration of data quality and use indicators in supervision tools and job aids (e.g., checklists)   **Intervention functionalities/components**   - Performance monitoring of health care provider(s) - Site-specific problem identification - On-the-job mentorship - Tailored improvement strategies | | |
|  |  |  |  |
|  |  |  |  |
|  |  |  |  |
|  |  |  |  |
|  |  |  |  |
|  |  |  |  |
|  |  |  |  |
|  |  |  |  |
|  |  |  |  |
| **Data use outcome** | **Evidence of the intervention's effect on data use** | **Strength of the evidence** | |
| **Intermediate outcomes** | **Improves data analysis, synthesis, and interpretation**   - Two reports with M&E data found that data management skills and practices of health facility staff improved between baseline and follow-up (e.g., ability to correctly calculate dropout rates, fill out monitoring charts, and properly archive data).^93,92^ | Low |  |
|  | **Improves data quality**   - One organizational-level survey and two reports with M&E data found improved data quality, congruency between data collection tools, and timelier reporting in HMIS.^86,92,93^ | Moderate |  |
|  | **Improves data availability**   - One RCT found a statistically significant increase (from 15.4% to 33.3%; p = 0.05) in the completeness of child vaccination records and no change in the control group (from 18.6% to 17.5%; p = o.69 ).^87^ | High |  |
| **Data use by health facilities** | **We are uncertain about the effect on data use by health facilities**   - One report with M&E data found an increase in the proportion of health facilities with documented evidence of data use(from 39% to 53% between rounds 1 and 2).60 However, a rapid organizational-level survey of the same intervention found that no health facilities reported implementing data use recommendations; rather, recommendations related to data management were implemented more often.^86^ | Very low |  |
| **Data use by health districts** | **We are uncertain about the effect on data use by health districts**   - One report with M&E data found an increase in the proportion of districts with documented evidence of data use (from 68% to 77% between rounds 1 and 2).60 However, a rapid organizational-level survey of the same intervention found minimal evidence of data use actions; instead, health districts were more likely to address recommendations related to data management and collection.^86^ | Very low |  |
| **Data use by national program** | **Uncertain**   - No studies were identified that reported this outcome. | No  evidence |  |

**Table 9 Training**

| **Hypothesized mechanisms** | **Contextual factors that affected how the intervention worked and intervention components hypothesized to support data use** | |
| --- | --- | --- |
| **Skills**  Strengthens skills in data collection. analysis, and interpretation  **Capability**  Builds capability to generate and use data to inform programmatic decisions  **Demand**  Increases the demand for timely, high­ quality data by improving data ­ related skills and demonstrating the value of data  **Data Quality**  Increases data quality by improving capabilities surrounding data | **Capability factors**   - Extent to which training is designed to address gaps related to M&E, epidemiology, health informatics, and surveillance - Extent to which training is reinforced by strategies such as applied group problem-solving, peer learning, and supervision   **Motivation factors**   - Extent to which training conveys the value of data, not just at higher levels of the health system but also at the facility level where data are produced   **Opportunity factors**   - Creation of new cadres of health workers responsible for M&E   **Intervention functionalities/components**   - Pretraining assessments to identify skill gaps - Applied learning component to reinforce training concepts | |
|  |  |  |
|  |  |  |
|  |  |  |
|  |  |  |
|  |  |  |
|  |  |  |
|  |  |  |
| **Data use outcome** | **Evidence of the intervention's effect on data use** | **Strength of the evidence** |
| **Intermediate outcomes** | **Improves analysis, synthesis, interpretation, and review of data**  *In the evidence from other health sectors:*   - One post-training assessment found increased confidence and capability in interpreting data and assessing the achievement of indicator targets.^94^ - One longitudinal evaluation of an intervention to create a new cadre of district M&E officers showed an increase in activities to strengthen data management, quality, reporting, and utilization for evidence-based planning.^97^ | Low |
| **Data use by health facilities** | **Improves the use of data at the health facility level**   - The Data for Decision Making Project in Cameroon provided anecdotal evidence of health officers using data to monitor disease burden and implementing immunization campaigns in response to an epidemic.^95^ | Low |
| **Data use by health districts** | **Improves the use of data at the district level**  *In the evidence from other health sectors:*   - Results from evaluation of the creation of a new cadre of district M&E personnel provided anecdotal, self-reported evidence of improved quality and use of data at the district level.^97^ | Low |
| **Data use by national program** | **Contributes to improvements in the use of data at the national level**   - Anecdotal evidence from the multi-country Data for Decision Making Project evaluation in Bolivia suggested improvements in data use. In Mexico, data on the health burden of tobacco use were used to advocate for, develop, and implement a tobacco-prevention policy.^95^ | Low |
